# Several new records, synonyms, and hybrid-origin of Chinese begonias

**DOI:** 10.3897/phytokeys.153.50805

**Published:** 2020-07-16

**Authors:** Dai-Ke Tian, Yan Xiao, Yan-Ci Li, Ke-Jian Yan

**Affiliations:** 1 Shanghai Chenshan Plant Science Research Center of Chinese Academy of Sciences, Shanghai Chenshan Botanical Garden, Shanghai 201602, China Shanghai Chenshan Plant Science Research Center of Chinese Academy of Sciences Shanghai China; 2 Shanghai Key Laboratory of Plant Functional Genomics and Resources, Shanghai 201602, China Shanghai Key Laboratory of Plant Functional Genomics and Resources Shanghai China; 3 Shanghai Normal University, Shanghai 200234, China hanghai Normal University Shanghai China; 4 Guangxi Institute of Traditional Chinese Medicine & Pharmaceutical Science, Nanning 530022, China Guangxi Institute of Traditional Chinese Medicine & Pharmaceutical Science Nanning China

**Keywords:** Begoniaceae, China, the Himalayas, natural hybrid, Nepal, stolon, taxonomy, Tibet

## Abstract

*Begonia* is a mega-genus with about 2500 species by most estimates, with China having over 210 accepted species. After field surveys, literature review and examination of herbarium specimens, some new taxa, new records, synonyms and the hybrid-origin of some taxa have been confirmed. Here, we report that *Begonia dioica* Buch.-Ham. ex D.Don and *B. flagellaris* Hara, both from Xizang (Tibet) are new to China; *Begonia lipingensis* Hance, *B. muliensis* T.T.Yu and *B. sizemoreae* Kiew are synonyms of *B. circumlobata* Hance, *B. taliensis* Gagnepain and *B. longiciliata* C.Y.Wu, respectively; and Begonia × lancangensis S.H.Huang and B. × malipoensis S.H.Huang & Y.M.Shui are natural hybrids.

## Introduction

*Begonia* is a mega-genus with an estimated 2500 species (Tian et al. 2018) and, so far, there are about 1978 known accepted species (Hughes et al. 2015–present). China has over 210 accepted names of *Begonia* and its southwest region is one of the distribution centres of this genus. In the past two years, ten *Begonia* species were newly described (Chen et al. 2018a, 2019; Ding et al. 2018; Li et al. 2018, 2019; He et al. 2019; Tian et al. 2019; Tong et al. 2019; Wang et al. 2019a) and many more are likely awaiting discovery and description in China. At the same time, several newly-recorded Chinese *Begonia* species (Yang et al. 2015; Wang et al. 2019b) and synonyms (Shui and Chen 2017; Chen et al. 2018b) have also recently been reported. With the support of the National Natural Science Foundation of China, we conducted many field surveys along with a review of literature and herbarium specimens related to Chinese *Begonia*. The diversity of Chinese *Begonia* is now better understood and several taxonomic issues have been resolved. Here, we report two new records, three new synonyms and two hybrid-origins of *Begonia* in China. Their conservation status was evaluated according to Guidelines for Using the International Union for Conservation of Nature (IUCN) Red List Categories and Criteria (v. 14) (IUCN Standards and Petitions Subcommittee 2019).

## New records

In September 2017, Daike Tian and his associates searched for wild begonias in Xizang (Tibet) of China. During this trip, two new records of stoloniferous begonias were discovered, namely *Begonia dioica* Buch.-Ham. ex D.Don (Don 1825) from Chentang town of Dingjie County and *B. flagellaris* H.Hara (Hara 1973) from Jilong town of Jilong County. Both species are distributed near the border between China and Nepal. At the time, *B. dioica* had only immature fruits with persistent tepals, while very few plants of *B. flagellaris* were still in bloom.

### Begonia dioica

Taxon classificationPlantaeCucurbitalesBegoniaceae

Buch.-Ham. ex D.Don [sect. Diploclinium]

2956B9CC-D9A3-5A4D-AC61-275A1A9F52FF

**Figure 1:**
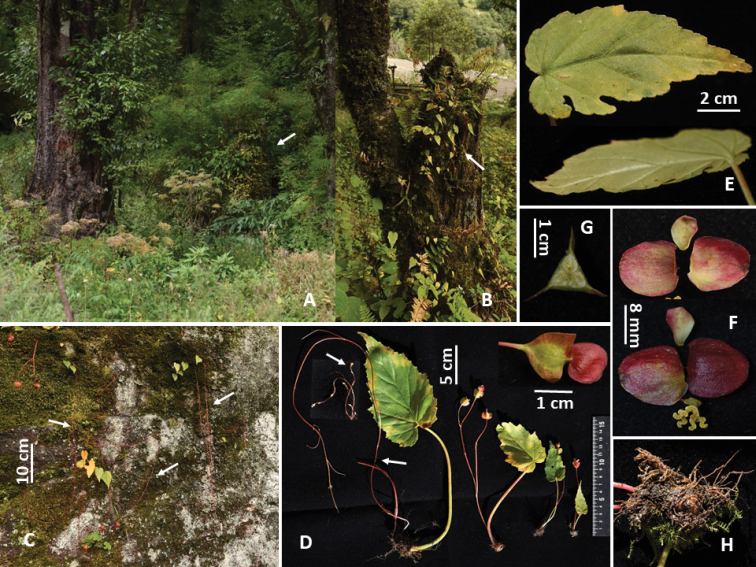
. Habitat and morphology of *Begonia dioica* (Photos by Daike Tian) **A, B** habitat (rock-moss surface and tree trunk, arrows indicate begonia plants) **C** individuals with long red stolons (arrows indicate stolons) **D** plants of different size and stolons with small whitish aerial bulbs (arrows indicate tiny bulbs) **E** leaves showing glabrous adaxial (upper) and abaxial (low) surfaces **F** female flowers with three tepals (upper: adaxial view, low: abaxial view) **G** cross-section of ovary with bilamellate axile placenta and three locules **H** tubers under moss.

Fig. 1 走茎秋海棠 (Chinese name)

Begonia dioica Buch.-Ham. ex D.Don [sect. Diploclinium] D. Don, *Prodr. Fl. Nepal*. 223. 1825: 223; R. Camfield & M. Hughes, *Eur. J. Taxon*. 396: 35. 2018.

#### Description.

Tuberous, creeping, stoloniferous, dioecious, deciduous herb, 3–11 cm high. All plant parts glabrous. Tubers 2–3 (1–2 old, one new). ***Stolon***: usually one to three developing from previous year’s tuber, red, slender, 5–60 cm long, 1–2 mm thick, usually unbranched, rarely branched or towards the apex with many fibre-like branches in large individuals, one to many tiny white aerial bulbs on stolon tips, gradually turning red after stolons touch moss or rock surface. ***Stipule***: lanceolate, 3–4 × 1–2 mm, glabrous, caduceus. ***Leaf***: 1 per plant, basal, petiole green to red, 1–22 cm long, 1.5–5 mm thick, adaxially shallowly grooved along the full length; lamina narrowly deltate-ovate, basifixed, symmetric, 2.5–17 × 1.5–10 cm, upper surface green, underside green, pink green or red, venation palmate, 8–9, green to red, adaxially impressed, abaxially prominent, tertiary even secondary veins invisible; base shallowly cordate, auricles non-overlapped, margin crenate to dentate or double serrate; apex acuminate. ***Inflorescence***: cymose, usually 1, terminal, 8–22 cm long, rachis pink to red, 6–10 cm long, 1–2 mm thick; peduncle branched up to three times, primary 5–10 cm long, secondary and tertiary 3–5 mm long, with 2–5 female flowers or 3–5 male flowers. ***Bract***: lanceolate 2–8 × 1–2 mm, caduceus. ***Male flower***: pedicel 10–25 mm long; tepals 4; outer tepals ovate-orbicular, 6–15 × 5–10 mm, pink to red, margin entire; inner tepals elliptic, 4–8 × 2–4 mm, white to pale pink; androecium with 15–20 stamens; filaments 1–2 mm long, unequal, fused at base into a short column; anther obovate, 1 mm long, dehiscing via short slits near the tip, not hooded, connective not extended. ***Female flower***: pedicel 12–30 mm long; bracteoles absent; tepals 3 (occasionally 2), persistent, outer two larger, elliptic-ovate, nearly equal, 6–15 × 6–10 mm, pink to red, inner one smaller, lanceolate, 6–7 × 3–5 mm, white to pink; ovary 3–locular, placentae bifid; styles 3, persistent, deeply forked once and spirally 1.5–2 circled. ***Fruit***: pendulous, capsule ellipsoid, 7–10 × 6–8 mm; wings 3, unequal or nearly equal, red or reddish-green, rounded-triangular, 2–6 × 7–12 mm, stalk red, 15–40 mm long, 0.8–1 mm thick.

#### Specimen collected from China.

**Xizang** (Tibet): Chentang Zhen of Dingjie Xian, 87°26'30.70"N, 27°50'54.11"E, alt. 2427 m, on rock surface and tree trunks. 19 Sept 2017, *Daike Tian*, *Yan Xiao and Zhu Lu TDK3306* (CHS).

#### Distribution and phenology.

Southern Xizang of China, northern Pakistan, northern India, Nepal and Bhutan; alt. 1350–2430 m; Flowering July to September, fruiting August to November.

#### Conservation status.

Least Concern (LC). *Begonia dioica* has numerous suitable habitats throughout its distribution range (Camfield and Hughes 2018). However, it should be considered as Critically Endangered (B2ab(v)) for China at the country level because only one population has been found so far and a continuing decline in the number of mature individuals is predicted due to road construction and other human’ activities.

#### Remarks.

Most of the individuals develop long stolons only from tubers formed in the previous year. The stolons are often branched in large individuals and the branch tops produce one to many tiny whitish bulbs, which grow larger as they touch the surface of a rock, tree trunk, soil or moss and then can develop into small plants in the second year. The tepals of female flowers are always persistent, even as the fruits mature.

### Begonia flagellaris

Taxon classificationPlantaeCucurbitalesBegoniaceae

Hara [sect. Diploclinium]

B8F108EA-A628-5C7A-A3C2-02D38236A1D1

**Figure 2.**
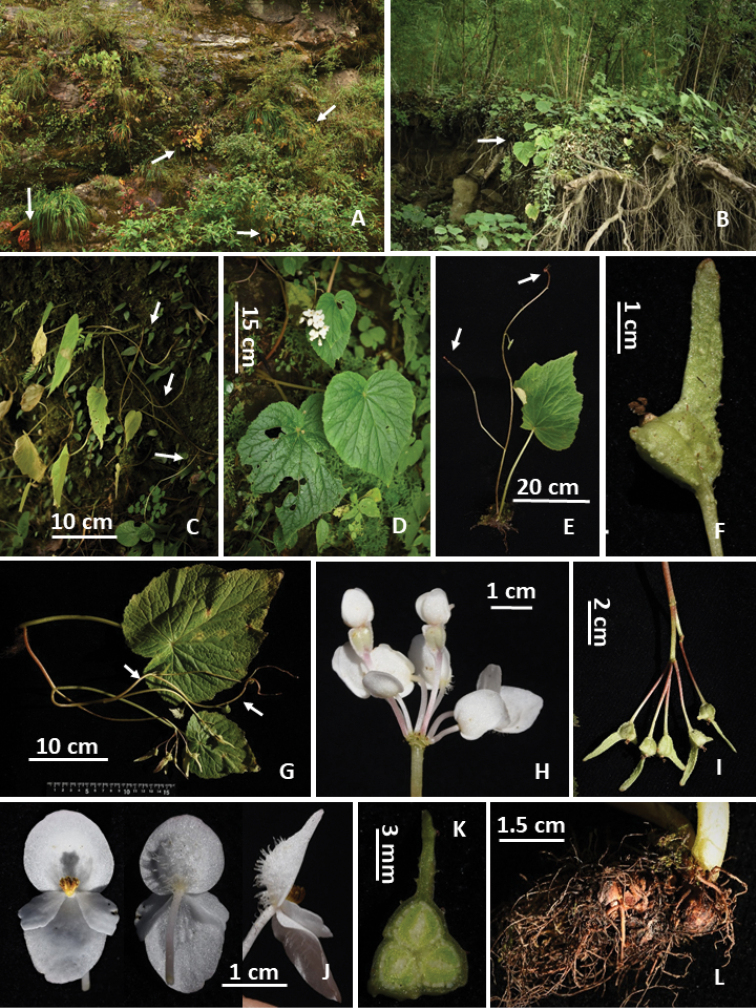
Habitat and morphology of *Begonia flagellaris* (Photos by Daike Tian) **A, B** habitat (rock hill or under bamboos, arrows indicate begonia plants) **C** individuals with long stolons **D** flowering plant **E** individual with aerial bulbs on stolon tips (arrows indicate tiny aerial bulbs) **F** fruit with extremely unequal wings **G** large individual with stolons (arrows indicate stolons) and fruits **H** simple umbellate inflorescence with white male and female flowers **I** infructescence **J** male flowers in front, dorsal and side views, respectively **K** cross-section of an ovary with the bilamellate axile placenta and three locules **L** underground tubers.

Fig. 2 鞭状秋海棠 (Chinese name)

Begonia flagellaris Hara [sect. Diploclinium] *J. Japan. Bot.* 48(12): 358–359, f. 3 (1973).

#### Description.

Tuberous, stoloniferous, dioecious, deciduous herb, 2–20 cm tall. Tubers usually 2–4 (1–3 old, red-brown, one new, white) connected, 3–15 mm diameter. ***Stolon***: developing from underground tubers or inflorescence; usually one per plant, green, unbranched to rarely branched, glabrous, slender, 10–50 cm long, 2–5 mm thick, aerial bulbs produced at stolon tips, 1–5 mm thick. ***Leaf***: usually one basal large and none to several smaller cauline (on stolons or peduncles), petiole green, 2–28 cm long, 1.5–7 mm thick, sparsely hairy; lamina basifixed, symmetric or nearly so, cordate, 2.5–26 × 1.2–28 cm, adaxial surface green, with short warty-base hairs, underside pale green, sparsely hairy; venation palmate, 9–11, green, adaxially impressed, abaxially prominent, base cordate, auricles non-overlapped to slightly overlapped, margin irregularly serrate to occasionally double serrate, rarely one to few shallowly lobed; apex acuminate. ***Inflorescence***: simple umbellate, 1–2 from the lower part of the stem, 6–20 cm long, rachis green to pink, 4–17 cm long, 2–3 mm thick; peduncle nearly erect, glabrous. ***Male flower***: white to pinkish, pedicel 14–28 mm long, 1 mm thick, top sparsely hairy; corolla 18–24 × 10–12 mm, tepals 4, outer 2, ovate, subequal, 7–12 × 7–11 mm, upper one centre thick and concaved, adaxially white hairy, up 1 mm long, less hairy on lower tepal; inner 2, glabrous, obovate to obovate-lanceolate, 7 × 4–5 mm; androecium leaning towards upper tepal, stamens 10–14, filaments free, about 1 mm long, anther elliptic, up to 1.5 mm long, 0.8 mm wide, apex obtuse. ***Female flower***: pedicel 20–35 mm long, 1 mm thick; tepals 5, unequal, glabrous; ovary hairy, 3-locular, placentae bifid; stigmas and styles 3. ***Fruit***: pendulous, capsule ellipsoid, 6–9 × 4.5–5 mm; wings 3, unequal, green, adaxially wing extremely long, narrowly triangular, 10–28 × 5–7 mm, lateral wings extremely narrow to nearly absent; stalk red at lower part, 24–40 mm long, 1 mm thick.

#### Specimen collected from China.

**Xizang**: Jilong Xian, Jilong Zhen, under bamboos, 85°21'12"N, 28°21'41"E, alt. 2030 m, 23 Sept 2017, *Daike Tian*, *Yan Xiao and Zhu Lu*, *TDK3343* (CHS); on steep slope under forest or rocky hill, 85°21'43"N, 28°21'48"E, alt. 2360 m, same date, *Daike Tian*, *Yan Xiao and Zhu Lu*, *TDK3344* (CHS).

#### Distribution and phenology.

**China**: Xizang, Jilong Xian, Jilong Zhen, border of China and Nepal; **Nepal**. Alt.1650–2900 m. Flowering August to September (early October), fruiting September to November.

#### Conservation status.

Near Threatened (NT). *Begonia flagellaris* is distributed in both Nepal and China, and there are many individual plants in each population. However, this species should be considered as Endangered (B1ab(iii)) for China because only two populations have so far been found and both are by the roadside.

#### Remarks.

Stolons develop from underground tubers or the top of inflorescence (usually on larger plants), with several small leaves. Hara (Hara 1973) compared the similarity between this species and *B. picta* J.E.Smith (Smith 1805); however, the two are quite different in appearance. *Begonia flagellaris* is mostly similar to *B. adscendens* C.B.Clarke (1890: 26), but differs mainly by having long stolons and more hairs on the outer tepals of the male flowers.

## New synonyms

### Begonia circumlobata

Taxon classificationPlantaeCucurbitalesBegoniaceae

Hance, J. Bot. 21: 203, 1883 (Hance, 1883)

D2EDF341-05EC-56D7-ADDB-A35C79038277

Fig. 3

-Begonia lipingensis Irmscher, *Mitt. Inst. Allg. Bot. Hamburg* 6: 353, 1927 (Irmscher 1927). syn. nov. Type: China, Kweitschou (Guizhou), Liping, alt. 600 m, 21 July 1917, *Handel-Mazzzetti 10909* (holotype: WU0038812, WU!; isotype: E00265121, E!)

#### Type.

China, Canton (Guangdong), 05 Oct 1881, *Rev. Benjamin Couch Henry s.n.* (BM000944652, BM!).

#### Note.

**Figure 3.**
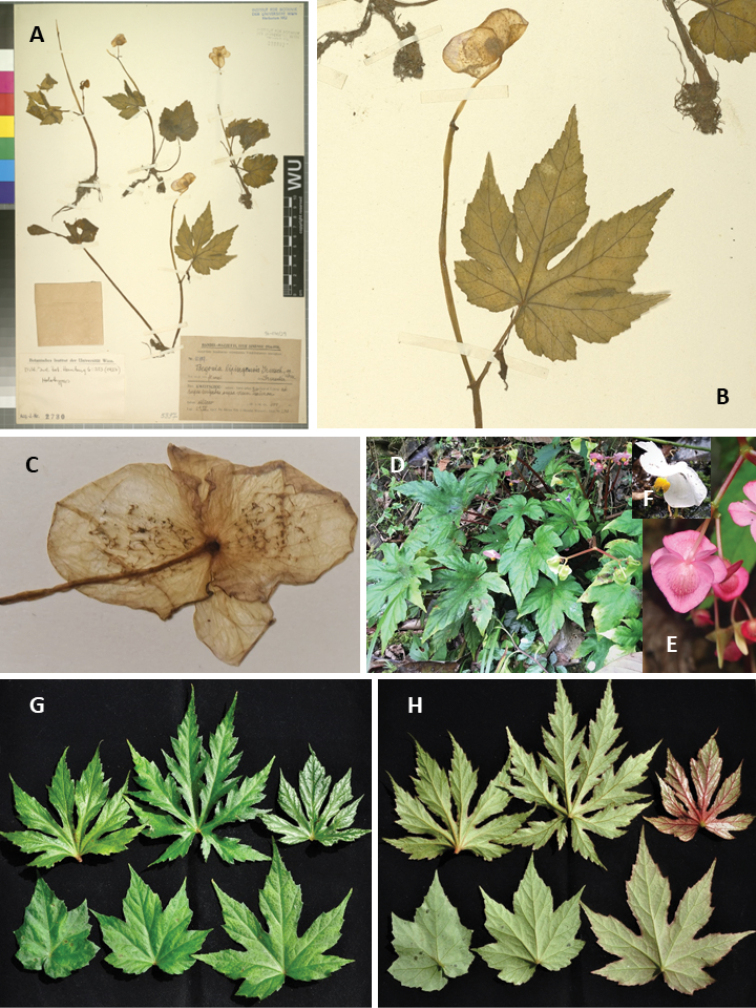
*Begonia lipingensis* and *B. circumlobata* (**E–H** photos by Daike Tian) **A–E***Begonia lipingensis*: **A** holotype (WU) (digitalised by Herbarium of Institut fur Botanik der Universitat Wien) **B** close-up view of type leaf **C** close-up of male flower from holotype, showing abaxial hairs on the middle of outer tepals **D** wild blooming plants **E, F** male flowers showing colour variation **G, H***Begonia circumlobata*: adaxial (**G**) and abaxial (**H**) views showing variations of leaf lobes and colour in a single small population.

*Begonia lipingensis* has been treated as a species differing from *B. circumlobata* in Flora Reipublicae Popularis Sinica (Ku 1999) and Flora of China (Gu et al. 2007). The species was based on small-sized mature plants that were collected from Liping County of Guizhou Province, China (Fig. 3A–C). However, the species could not be separated when comparing the specimens and living plants in the wild. Many specimens stored in herbaria of China or other countries were identified with both names. After our careful review of type specimens and comprehensive field surveys on a large number of populations of both entities, it was confirmed that no differences could be found between them. Since *B. lipingensis* was described later, it should be considered as a synonym of *B. circumlobata*.

*Begonia circumlobata* displays significant variation in plant size, morphology of leaves, and flowers (Fig. 3E–H). The leaves may be shallowly to very deeply lobed in the same population, even for the same individual and very few of the plants are shallowly double-lobed like *B. jinyunensis* C.I Peng, B.Ding & Q.Wang (Ding et al. 2014) (Fig. 3G, H). The leaves of most plants are pure green on two sides, while others may have deep green leaves with abxially red surface. Occasionally, variegated plants with white-spotted leaves could be seen in some populations such as in Huangsang National Nature Reserve of Suining, Hunan Province. Flowers can be white, whitish-pink, pink or nearly red (Fig. 3D, E).

#### Distribution and phenology.

**Figure 4.**
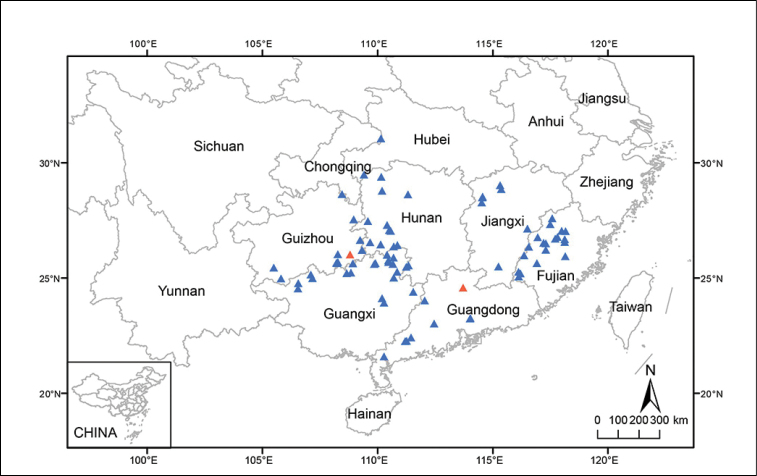
Distribution map of *B. circumlobata* (including syn. *B. lipingensis*) Triangles show distribution based on specimens and field surveys and red triangles indicate type locality of *B. circumlobata* (Guangdong) and *B. lipingensis* (Guizhou), respectively.

*Begonia circumlobata* is widely distributed in at least seven provinces of China, from western Hubei to Hunan, Jiangxi, Fujian, Guangdong, Guangxi and Guizhou, growing on flat areas, steep slopes or rock surfaces along or near stream and valley. Alt. 200–1230 m (Fig. 4). Flowering June to September, fruiting July to October.

#### Conservation status.

Least Concern (LC) due to wide distribution and usually large populations. However, in some places, a small number of plants with variegated leaves (adaxially white spots) have high value as ornamentals. Therefore, these variegated individuals may be over-collected by humans.

#### Remarks.

*Begonia circumlobata* has sparsely hairy leaf blades and outer tepals of male flowers (Fig. 3C). It is most similar to *B. jinyunensis* and *B. laminariae*, particularly in the morphology of its leaves, flowers and fruits. *Begonia circumlobata* is also easily confused with some individuals of *B. pedatifida* Lév. (Léveillé 1909), particularly when examining herbarium specimens. Unpublished morphological and molecular data (Tian et al.) suggests that *B. jinyunensis* should be treated as a subspecies of *B. circumlobata*. At the same time, a study is ongoing concerning the taxonomic relationship of *B. circumlobata*, *B. laminariae* Irmsch. (Irmscher 1951) and *B. pedatifida*. Since plants with intermediate morphology amongst these three taxa exist in the wild, it appears that gene flow or natural hybridisation might occur between them.

### Begonia longiciliata

Taxon classificationPlantaeCucurbitalesBegoniaceae

C.Y.Wu, Acta Phytotaxon. Sinica 33(3): 251, 1995 (Wu and Ku 1995)

F0973001-2A2E-538D-AE02-E647A5CE0D70

Fig. 5

-Begonia sizemoreae Kiew, Gard. Bull. Singap. 54(6): 95–100, 2004. syn. nov. Type: Vietnam, Ha Tay Province: Ba Vi National Park, no date, *R. Kiew* 5304 (holotype: SING!; isotype: HN!).

#### Type.

China, Guizou: Anlong, alt. 990 m, 14 May 1960, *Guizhou Exped. 5117* (holotype: KUN!; isotye: PE!).

#### Note.

**Figure 5.**
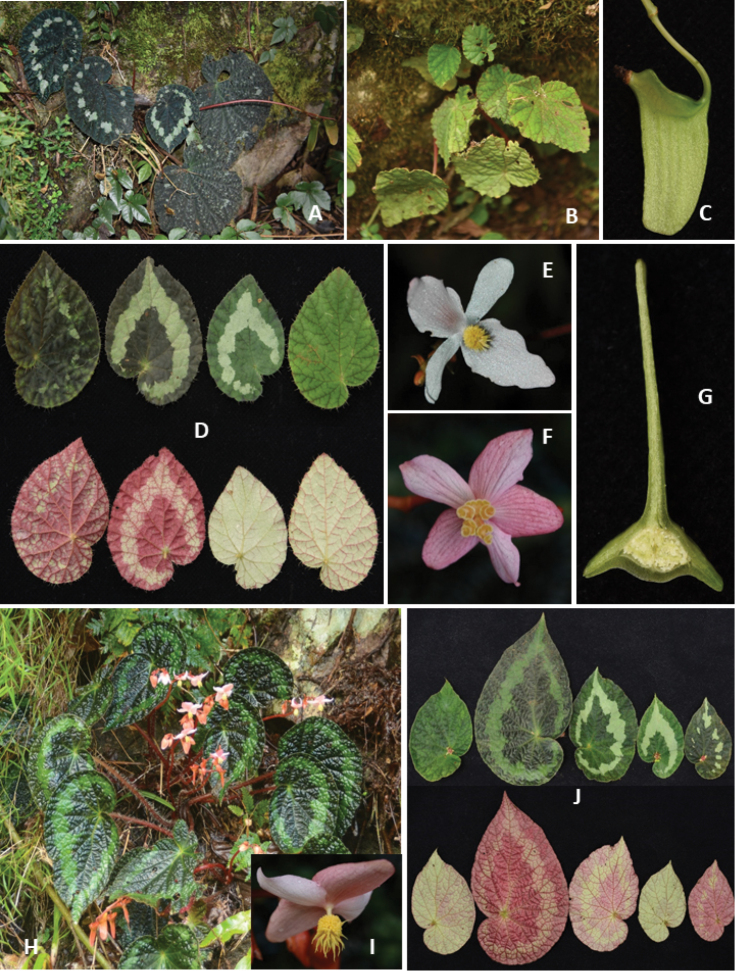
Morphological variation of *Begonia longiciliata* in China(Photos by Daike Tian) **A–E** population from Guizhou Province: **A** individual with dark green leaves and white variegation (near white ring or isolated white spots) **B** pure green-leaved individual **C** fruit with one long wing and two short wings **D** comparison of adaxial (upper) and abaxial (low) views of leaf variation in colour and variegation **E** male flower (deep-pink one not shown) **F–J** population from Yunnan province: **F** female flower, showing pink variant **G** cross-section of ovary showing two locules and bilamillate placenta **H** dark-green leaved individual with a light-green ring band **I** male flower showing very long anther in upper portion of androecium **J** comparison of adaxial (upper five leaves) and abaxial (lower five leaves), showing differences in leaf colour and variegation of different individuals.

**Figure 6.**
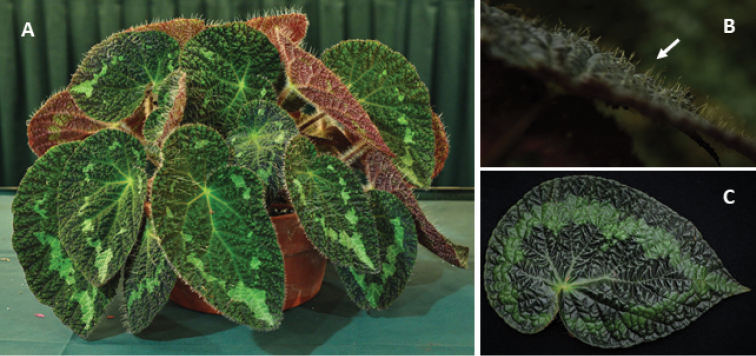
Comparison on hairy and glabrous adaxial leaf surface of *B. longiciliata***A** plant with hairs (cultivated as *Begonia* U388, American Begonia Society Conference 2012) **B** Guizhou population with hairs (arrow direction) **C** Yunnan population with glabrous adaxial leaf surface.(Photos by Daike Tian).

**Figure 7.**
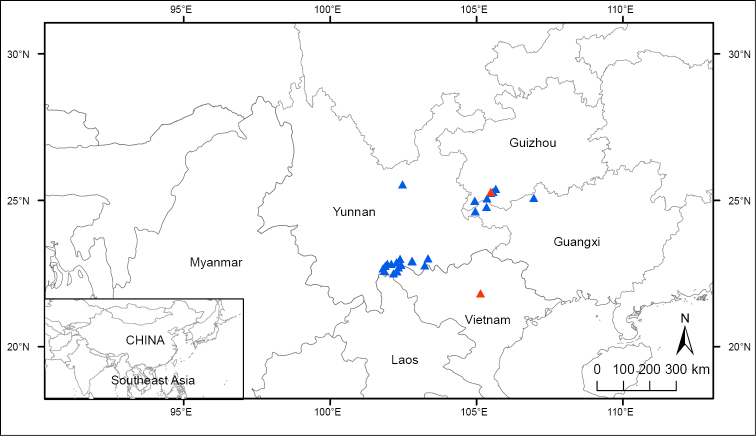
Distribution of *Begonia longiciliata* (including syn. *B. sizemoreae*) Triangles show distribution based on specimens and field survey and red triangles indicate type locality of *B. longiciliata* (Guizhou, China) and *B. sizemoreae* (Bavi, Vietnam), respectively.

*Begonia longiciliata* (Fig. 5) is mostly close to *B. rex* Putz. (Putzey, 1857), but differs mainly by its narrower tepals of both its male and female flowers and longer anthers (up to 4 mm long) with acuminate tips that occur towards the apex of the androecium (Fig. 5I). Notably, it has large variation in leaf colour, variegation patterns and flower colour varying from white, pink to even nearly red (Fig. 5). *Begonia rex* is only found in India, while *B. longiciliata* has a wide distribution from Guizhou, Guangxi and southern Yunnan of China, to the north of both Laos and Vietnam (Fig. 7). The name *longiciliata* probably refers to the long fibre-like hairs found on the adaxial leaf surface in some populations of this taxon in Guizhou Province (type locality) (Fig. 6B), but most populations have glabrous adaxial leaf surface or nearly so (Fig. 6C) particularly in Yunnan Province. It is also similar to *B. annulata* K.Koch (Koch, 1837) in leaf morphology, but can be easily separated by the latter’s hairy (vs. glabrous) tepals of both male and female flowers and fruits (Camfield and Hughes 2018).

*Begonia longiciliata* has been wrongly treated as *B. rex* in China (Ku 1999; Gu et al. 2007) and was even treated as a new species (*B. sizemoreae*) in 2004 (Kiew 2004), based on a type specimen collected in Ba Vi National Park in northern Vietnam. The material from China, Vietnam and Loas is mostly similar; the imaged type plants of *B. sizemoreae* from northern Vietnam are nearly identical to plants of *B. longiciliata* from China; *B. longicilata* and *B. sizemoreae* are distributed mostly along the China-Vietnam boundary regions; recently, the species was recorded as *B. sizemoreae* in northern Laos (Ding et al. 2020); no stably different key characteristics are found between *B. longicilata* and *B. sizemoreae*. Therefore, *B. sizemoreae* is considered by us as a synonym of *B. longicilata*.

#### Specimens examined.

**China: Yunnan**, **Jiangcheng**: Kukazai Qushui, 14 Dec. 1991, Guoda Tao 49032; Tukahe, 18 Dec 1991, Guoda Tao 47818, 49127 (HITBC); Jiahe, 23 Sept 2015, Daike Tian et al. TDK2659 (CHS). Pingzhangzhai, Pingzhang village, Jiahe, 30 Oct 2012, Jiangcheng Survey Team 5308260564 (IMDY); Jiahe to Xiaoheijiang, 21 Oct 2011, Daike Tian et al. TDK252, TDK253 (CHS); Jiangcheng county to Daheishan, 21 Oct 2011, Daike Tian et al. TDK257 (CHS). **Jinping**: Riverside, 22 Oct 2008, Xiaohua Jin 9467 (PE); Laomeng, 22 Nov 2007, Yumin Shui et al. 80105 (KUN). **Lüchun**: Laomenghe, 22 May 1974, Lüchun Team 1092 (KUN); Huanglianshan, 30 Oct 1995, Sugong Wu et al. 379 (KUN), 31 Oct 1995, Sugong Wu et al. 3354 (KUN), Sugong Wu et al. 3354 (PE), 01 Nov 1995, Sugong Wu et al. 2609 (KUN); Xiaohejiang, 18 Oct 2000, Yumin Shui & Wenhong Chen 13132, 13797 (KUN); Erpu to Banpo, 22 Oct 2000, Yumin Shui & Wenhong Chen 13620, 13696 (KUN); Erpu to Dapu, 23 Oct 2000, Yumin Shui & Wenhong Chen 14138 (KUN); 24 Oct 2000, Yumin Shui & Wenhong Chen 13848 (KUN); Xinzhai, Erpu, 03 Nov 2007, Yumin Shui et al. 72970 (KUN); Shiyazi, Daheishan, 22 Nov. 2011, Jianghai He et al. HLS0353 (KUN); Lüchun county to Manhao of Gejiu county, 25 Aug 2013, Daike Tian et al. TDK1281, TDK1283 (CHS); Daheishan, 23 Sept 2015, Daike Tian et al. TDK2661 (CHS); Dashuigou, 23 Sept 2015, Daike Tian et al. TDK2663 (CHS); Cheli of Pinghe, 23 Sept 2015, Daike Tian et al. TDK2680 (CHS); Xiaoheijiang, Xinzhai of Pinghe, 24 Sept 2015, Daike Tian et al. TDK2683, 2685 (CHS). **Luquan**: Mayu, 30 Oct 1995, Sugong Wu et al. 379 (PE). **Mengla**: Xishuangbanna Tropical Botanical Garden, Menglun, 21 Sept 2015, Daike Tian et al. TDK2629 (CHS) (cultivated). **Guangxi**, **Longlin**: Jinzhongshan, 23 May 1977, Zhou Fakai 3-0701 (GXMI); Same locality, 24 Sept 1984, Chinese Medicine Team 0185 (GXMI). **Tian’e**: Xiangyang, 01 May 1978, Tian’e Team 4-6-0255 (GXMI). **Guizhou**, **Anlong**: Huali of Tingya, 14 May 1960, Zhisong Zhang & Yongtian Zhang 3320 (PE); Xiaojiatang, Lishu village of Dushan, 15 Oct 2017, Daike Tian et al. TDK3473 (CHS); Xiaoanhe, Pojing of Dushan, 15 Oct 2017, Daike Tian et al. TDK3474 (CHS). **Xingyi**: Daojiao, Gongqiao of Zerong, 14 Oct 2017, Daike Tian et al. TDK3460 (CHS). **Zhenfeng**: 19 Sept 1936, Shiwei Deng 90987 (IBSC). **Unknown county**: Feb 1921, M. Cavalerie, unknown collection no. (P06841311) (P); Oct 1917, M. Esquirol, unknown collection no. (P05495115) (P).

**Laos**: Phongsaly, Tan et al. L0559 (HITBC) (Ding et al. 2020).

**Vietnam**: Ba Vi National Park, Ha Tay province, R. Kiew 5304 (SING, HN); Tonkin (Mountain Bavi), Dec 1887, B. Balansa 3765 (P); Tonkin, 29 Apr 1936, M. Polane 25811 (P).

#### Distribution and phenology.

**China: Guangxi** (Longlin, Tian’e), **Guizhou** (Anlong, Xingyi, Zhenfeng), **Yunnan** (Jiangcheng, Jinping, Lüchun, Luquan); **Laos** (Phongsaly); **Vietnam** (Ba Vi) (Fig. 7). Alt. 300–1300 m. Flowering May to November, fruiting June to December.

#### Conservation status.

Near Threatened (NT). *Begonia longiciliata* has a relatively broad distribution, particularly in the borders of China, Laos and Vietnam (Fig. 7); however, the size of most populations is small and the habitats are fragmented. Its distribution range is extremely narrow in both Guangxi and Guizhou provinces of China. Several populations exist with less than 20 or even 10 individuals. In these two provinces, the population size continues to decrease, with very little seedling recruitment, due to habitat deterioration and disturbance from agricultural activities. This species also needs an environment that has a high level of humidity to survive well. Additionally, because of its beautiful foliage, wild plants are at risk of overharvesting, therefore, it should be considered Vulnerable (B2ab(iv)) in China.

#### Remarks.

*Begonia longiciliata* has been treated as a synonym of *B. rex* for a long time in China (Ku 1999; Gu et al. 2007). Several horticultural cultivars have been produced by crossing it with other *Begonia* taxa at Kunming Botanical Garden of China (Tian et al. 2001, 2002). However, *B. rex* has not been collected or observed in the wild in China. It was recorded in several locations from Arunachal Pradesh (Camfield and Hughes 2018) (called southern Tibet by China), a currently China-India disputed region. In addition, *B. longiciliata* was previously cultivated under the code U3888 (with long hairs on adaxial leaf surface, Fig. 6A) by the American Begonia Society and these cultivated plants were correctly identified as *B. longiciliata* by Golding (Golding 2004), but were later treated by other researchers as *B. rex* (Ku 1999; Gu et al. 2007) or *B. sizemoreae* (Tebbitt 2005). *Begonia longiciliata* is officially established, based on our extensive field surveys, literature review, specimen examination and the observation of plants in cultivation. According to their morphological similarity and adjacent distribution, *B. longiciliata* and *B. rex* are similar to each other and they may have differentiated possibly due to geographic isolation. Further investigation is needed on their relationship and whether it is more reasonable to treat *B. longiciliata* as a subspecies or variety of *B. rex*.

### Begonia taliensis

Taxon classificationPlantaeCucurbitalesBegoniaceae

Gagnepain, Bull. Mus. Natl. Hist. Nat. 15: 279, 1919

73DDCDAE-4C43-52E1-8F19-80C364D0C4C3

Fig. 8

-Begonia muliensis T.T.Yu, *Bull. Fan. Mem. Inst. Biol.*, 1:119, 1948 (Yu 1948). syn. nov. Type: China, Sichuan: no locality data, no date, *T.T. Yü*, *14024* (A!)

#### Type.

China,Yunnan: Tali (Dali), 4 Sept. 1883, *J.M. Delavay 220* (Lectotype, P!, designated here).

#### Note.

**Figure 8.**
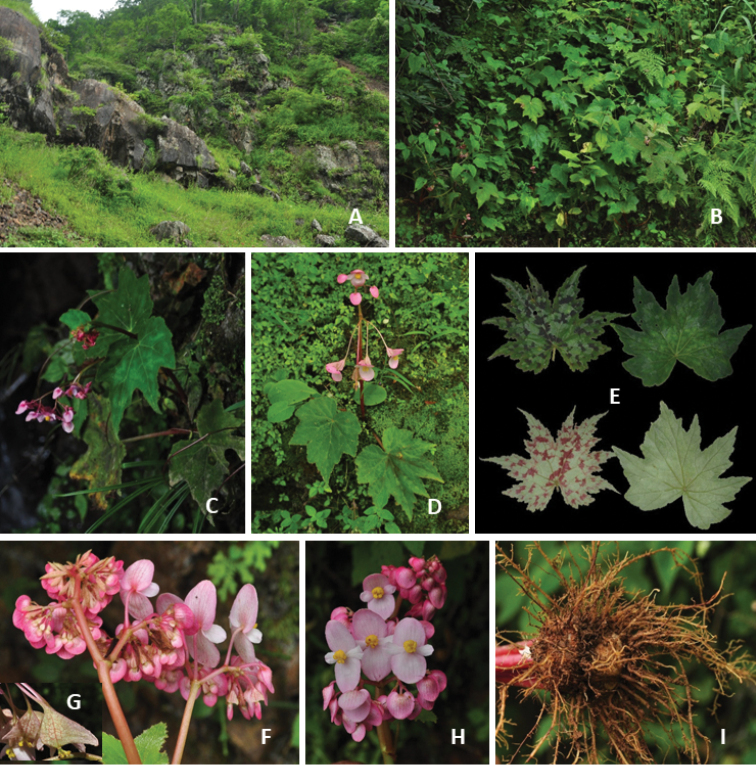
Habitat and morphology of *B. taliensis* (Photos by Daike Tian) **A** habitat **B** population with pure-green leaves **C, D** blooming individuals with variegated leaves **E** comparison of variegated and solid green-leaved individuals (adaxially and abaxially views) **F–H** inflorescence of large individuals and young fruits with red lines (**G**) **I** underground tubers (usually 2–3 connected) with numerous roots.

**Figure 9.**
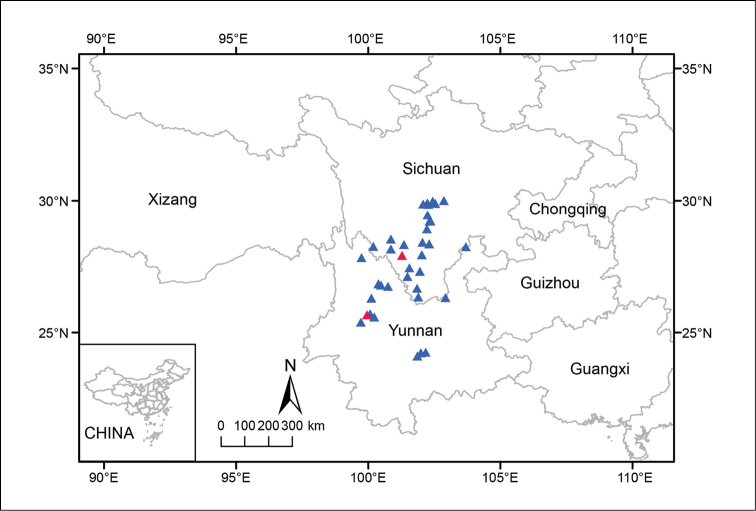
Distribution map of *Begonia taliensis* (syn. *B. muliensis*) Triangles show distribution sites, based on specimens and field surveys and red triangles indicate type locality of *B. muliensis* (Muli of Sichuan) and *B. taliensis* (Dali of Yunnan), respectively.

**Figure 10.**
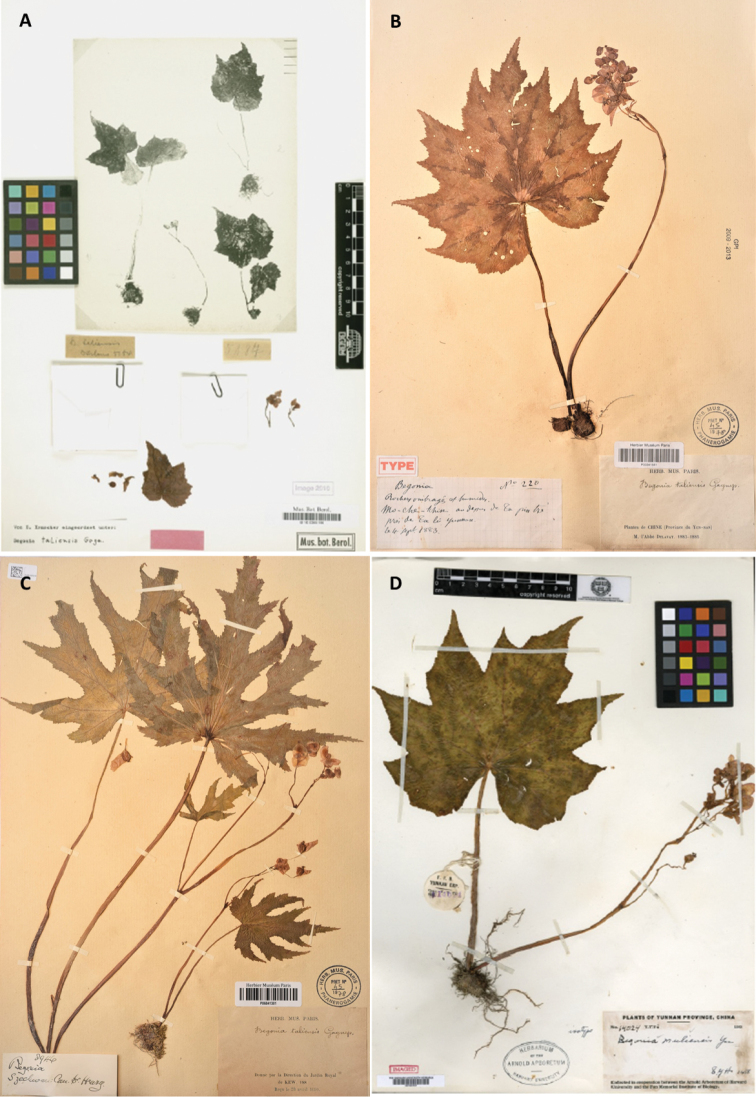
Comparison of types of *Begonia taliensis* (**A–C**) and *B. muliensis* (**D**) **A** Ducloux No. 5184 (Yunnan) **B** Delavay No. 220 (Yunnan) **C** Henry 8946 (Sichuan) **D** T.T. Yü #14024 (Sichuan) (**A** accessed JSTOR and imaged by Botanical Museum Berlin-Dahlem **B, C.** Photos by Daike Tian at Herbarium Museum of Paris **D** accessed JSTOR, Imaged by Herbarium of the Arnold Arboretum, Harvard University).

*Begonia taliensis* is relatively widely distributed in many counties from Yunnan Province to Sichuan Province in China (Fig. 9). It exhibits considerable variation in size of plant, leaf and inflorescence, shape of lobes, leaf colour and blade variegation patterns (Fig. 8). However, its net-like pattern of red lines on the fruits is a stable character (Fig. 10F, left corner). There is no distinction in distribution and morphology between it and *B. muliensis* (isotype, Fig. 10D) (HU). In addition, when *B. taliensis* was described and published, the author (Gagnepain 1919) cited three collections of specimens: Ducloux 5184 (B), Delavay 220 (P), and Henry 8946 (P) (Fig. 10). Unfortunately, he did not assign a type specimen. Of these, Ducloux 5184 and Delavay 220 were collected from Dali in Yunnan Province and both are significantly different only in plant size. However, Henry 8946 was collected from somewhere (possibly near Kangding County) in Sichuan Province. Dali and Kangding are at least 600 km apart. In fact, Henry 8946 is a specimen of *B. imitans* Irmsch. (1939: 51) (Fig. 10C) (lobed over 2/3). The relationship between *B. taliensis* and *B. imitans* remains unknown and further study is undergoing. Based on our literature review, field survey and type specimens, Delavay 220 is designated here as the lectotype of *B. taliensis* and Dulcoux 5184 as its syntype. The syntype Henry 8946 belongs to another species and should not be considered for future nomenclatural decisions.

#### Distribution and phenology.

**China: Sichuan** (Daocheng, Dechang, Luding, Meigu, Mianning, Muli, Panzhihua, Shimian, Tianquan, Yanbian, Xide); **Yunnan** (Dali, Eyuan, Heqing, Lijiang, Yangbi, Yongsheng, Zhongdian) (Fig. 9). Alt. 1000–3200 m. Flowering July to October, fruiting August to November.

#### Conservation status.

*Begonia taliensis* has a relatively-wide distribution (recorded or observed in nearly 20 counties of two provinces in China, Fig. 8), but the size of most populations is usually small. In addition, many of its distribution sites are near roadsides; therefore, the habitats could be easily disturbed by human activities. Additionally, this species is used as an ornamental due to its beautiful foliage and flowers or as a vegetable by local residents (Yang et al. 2018). Continuous human collection for different purposes may cause a decrease in population size and individual numbers. Therefore, its conservation status should be currently considered Near Threatened (NT).

#### Remarks.

The leaf colour of *B. taliensis* varies amongst populations and occasionally even amongst the individuals of a small population. The plants usually have leaves with abaxially purple-red blotches. Sometimes, a few plants or even all individuals of a small population are pure green in leaf colour. The leaf could be shallowly to 1/2 deeply lobed (vs. over 2/3 deeply lobed for *B. imitans*) depending on plant size or distribution site. The flower number ranges from around 10 for a small flowering individual to over 100 for a large one.

## Hybrid-origin taxa

Natural hybridisation is very common in *Begonia* and 50 populations of 31 natural hybrids involving 29 species have been recorded in Chinese wild begonias (Tian et al. 2017). Based on morphological and molecular analysis (Tian et al. 2017), two previously published species are considered to be of hybrid origin: *B. lancangensis* S.H.Huang (Shui and Huang 1999: 13) and *B. malipoensis* S.H.Huang & Y.M.Shui (Huang and Shui 1994). Therefore, these two species are formally recognised here as hybrids.

### Begonia × langcangensis

Taxon classificationPlantaeCucurbitalesBegoniaceae

S.H.Huang

BFA66D1F-8910-51FE-97C8-14DCA3BE4040

Fig. 11A, B

-Begonia langcangensis S.H.Huang, *Acta Bot. Yunnanica* 21:13, 1999; S.H. Huang & Y.M. Shui in C.Y. Wu (ed.), *Fl Yunnan* 12: 230, 2006; T.C. Ku et al. in C.Y. Wu & P.H. Raven (eds), *Fl. China* 13: 181, 2007.

#### Note.

**Figure 11.**
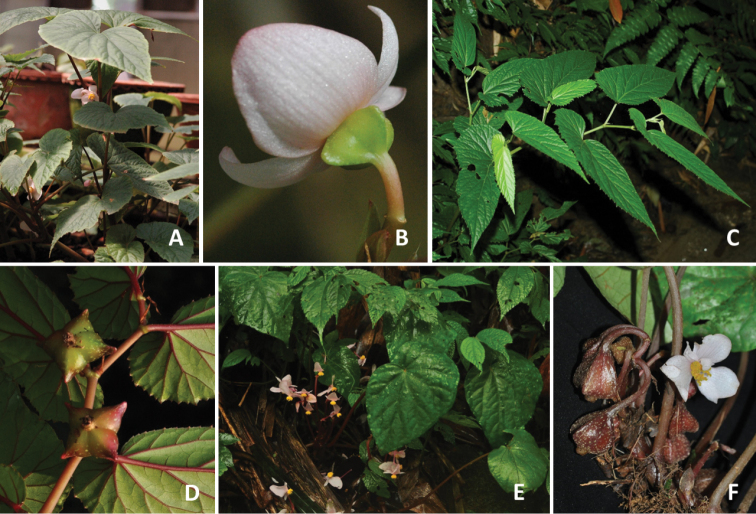
Begonia × lancangensis and its parents (*B. acetosella* and *B. handelii*) (Photos by Daike Tian) **A, B** male plant and female flower of B. × lancangensis**C, D** plant and fruits of *B. acetosella***E, F** male and female flowering plants of *B. handelii*.

*Begonia langcangensis* was described and published in 1999 and its type collection was made from Fazhan He of Lancang County in Yunnan Province. Since then, no additional specimens have been collected. During our field surveys in 2010 and 2017, respectively, we did not find any plants of this taxon in the type locality and only observed *B. acetosella* Craib (Craib 1912: 347) (Fig. 11C, D), *B. handelii* Irmsch. (Irmscher 1921) (Fig. 11E, F) and *B. palmata* D.Don (Don 1825). Based on the intermediate morphology of *B. langcangensis* and the overlapping distributions of *B. acetosella* and *B. handelii*, it is hypothesised that *B. langcangensis* is very likely a natural hybrid of these two species. To further investigate this, by artificially crossing *B. acetosella* and *B. handelii*, we produced, at Kunming Botanical Garden, a hybrid that was morphologically almost the same as *B. langcangensis* (Fig. 11A, B). Therefore, we confirmed that *B. langcangensis* is a natural hybrid. It is very similar to the hybrid (unpublished) in the same section of *B. acetosella* × B. silletensis subsp. mengyanensis Tebbitt & K.Y.Guan (Tebbitt and Guan 2002), which has hairy stems and petioles and larger leaves (Tian et al. 2017).

#### Distribution and phenology.

**China**: Yunnan, Lancang, only seen in type locality, alt. 1600 m; **Laos**: Luang Namtha Province, Nam Ha National Biodiversity Conservation Area, Near Na Lun Village, alt. 687 m (Ding et al. 2020). Flowering March to May, fruiting April to July.

#### Conservation status.

Regionally Extinct (RE). The living plants of Begonia × lancangensis have not been found in the type locality during field surveys after its first description. Recently, however, other researchers found wild plants in Laos (Ding et al. 2020).

#### Remarks.

Like *B. acetosella*, *B. handelii* and *B. silletensis* C.B.Clarke (Clarke 1879), dioecious Begonia × lancangensis has berry-like fruits and was previously classified in section Sphenanthera, but has recently been integrated into section Platycentrum (Moonlight et al. 2018). In the wild, *B. acetosella*, *B. handelii* and *B. silletensis* often have overlapping distributions, meaning natural cross fertilisation is possible due to their overlapping flowering periods. The hybrid plants are usually very few and, therefore, rarely observed, due to a low chance of a natural cross. Natural crossings may generate new hybrids in the future.

### Begonia × malipoensis

Taxon classificationPlantaeCucurbitalesBegoniaceae

S.H.Huang & Y.M.Shui

91819DD1-27A1-5BDA-9731-4C59F6A1741F

Fig. 12

-Begonia malipoensis S.H.Huang & Y.M.Shui, *Acta Bot. Yunnanica* 16:333, 1994.

#### Note.

**Figure 12.**
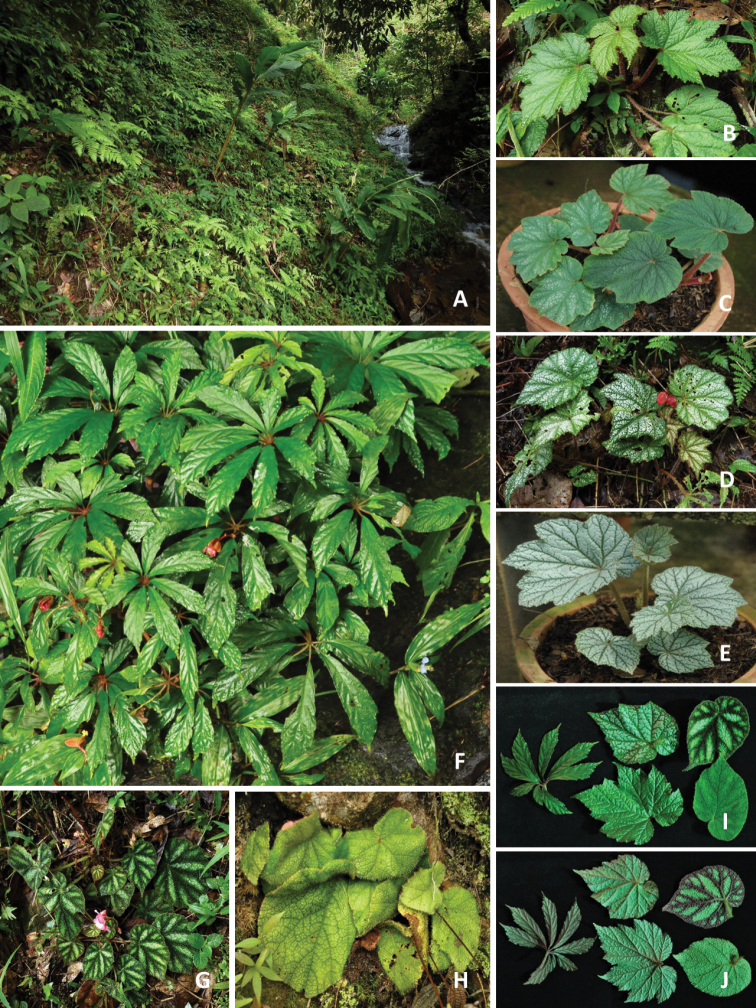
Begonia × malipoensis and its parents (*B. hemsleyana* and *B. versicolor*) (Photos by Daike Tian) **A** habitat of a natural hybrid zone of *B. versicolor* × *B. hemsleyana***B–E** variation of B. × malipoensis**F***B. hemsleyana***G, H***B. versicolor* with variegated and pure green leaves **I, J** comparison of B. × malipoensis (middle two leaves) and its parents *B. hemsleyana* (left) and *B. versicolor* (right two leaves) (**I** adaxial view **J** abaxial view).

*Begonia malipoensis* was described for the first time in 1994 and its type locality is Douchidian of Malipo Xian, Yunnan Province (Huang and Shui 1994). In the wild, it grows closely with *B. hemsleyana* Hook.f. (Curtis et al. 1899) (Fig. 12F) and *B. versicolor* Irmsch (Irmscher 1939) (Fig. 12E, F). Later, Daike Tian (Tian 1999) conducted field surveys on the diversity of *B. versicolor* in southeastern Yunnan and found a few plants of *B. malipoensis* in the same locality and at Daweishan National Nature Reserve of Pingbian County, Yunnan Province. Based on the very limited number of individuals and intermediate morphology between *B. hemsleyana* and *B. versicolor*, *B. malipoensis* is considered a natural hybrid and this supposition was confirmed by artificial cross experiments (Tian 1999). From natural hybrids, one type, with densely white-spotted leaves, was selected as a new cultivar, B. × malipoensis ‘White Snow’ (Tian et al. 2001). The hybrid status of B. × malipoensis was further supported by molecular evidence (Tian et al. 2018).

The hybrid B. × malipoensis is derived from either *B. hemsleyana* × *B. versicolor* or B. versicolor × hemsleyana. No significant differences were observed in the hybrid when either *B. hemsleyana* or *B. versicolor* acts as the mother plant. However, based on a presumed closer distance with mother plants and molecular data (Tian et al. 2017), more wild hybrids occurred with *B. hemsleyana* as a mother plant in Malipo county, while more with *B. versicolor* as mother plant were observed in a hybrid zone in Pingbian county.

#### Distribution and phenology.

B. × malipoensis has only been seen in Malipo and Pingbian counties in Yunnan Province. Flowering June to July, fruiting July to September.

#### Conservation status.

Critically Endangered (C2a(i)). It is extremely narrowly distributed with less than 100 mature individuals and can only be found in the hybrid zones of two locations in China. The hybrid plants are continuously collected by horticultural researchers or plant enthusiasts, mainly for ornamental purposes.

#### Remarks.

B. × malipoensis is difficult to bloom under ex-situ cultivation. When the seeds from an artificial cross between *B. hemsleyana* and *B. versicolor* were sown, the plants produced had various types of leaf colour and colour patterns (Tian 1999).

## Supplementary Material

XML Treatment for Begonia dioica

XML Treatment for Begonia flagellaris

XML Treatment for Begonia circumlobata

XML Treatment for Begonia longiciliata

XML Treatment for Begonia taliensis

XML Treatment for Begonia × langcangensis

XML Treatment for Begonia × malipoensis

## References

[B1] Camfield R Hughes M (2018) A revision and one new species of *Begonia* L. (Begoniaceae, Cucurbitales) in Northeast India. European Journal of Taxonomy 396: 1–116. 10.5852/ejt.2018.396

[B2] Chen WH Radbouchoom S Nguyen HQ Nguyen HT Nguyen KS Shui YM (2018a) Seven new species of *Begonia* (Begoniaceae) in northern Vietnam and southern China. PhytoKeys 94: 65–85. 10.3897/phytokeys.94.23248 PMC5799780 29416422

[B3] Chen WH Radbouchoom S Nguyen HQ Phutthai T Leonid VA Shui YM (2018b) Reassessment of *Begonia arboreta* and *B. sonlaensis* (Begoniaceae) based on field observation and type examination. Phytotaxa 381(1): 132–140. 10.11646/phytotaxa.381.1.17

[B4] Chen WH Guo SW Radbouchoom S Dong WK Wang ZX Xi HH Shui YM (2019) A new berry-fruited species of *Begonia* (Begoniaceae) from Xizang (Tibet). Phytotaxa 407(1): 29–35. 10.11646/phytotaxa.407.1.5

[B5] Clarke CB (1879) Begoniaceae. In: Hooker JD (Ed.) Flora of British India 2: 635–636. https://biodiversitylibrary.org/page/37371521[accessed 20.01.2020]

[B6] Clarke CB (1890) On the plants of Kohima and Muneypore. Journal of the Linnean Society. Botany 25: 1–107. 10.1111/j.1095-8339.1889.tb00793.x [accessed 20.01.2020]

[B7] Craib WG (1912) Contributions to the Flora of Siam. Bulletin of Miscellaneous Information, Kew (3): 144–154. 10.2307/4104569

[B8] Curtis W Hooker JD Hooker WJ Prain D Stapf O (1899) Curtis’s Botanical Magazine 125: Tab. 7685. https://www.biodiversitylibrary.org/item/14253#page/212/mode/1up [accessed 20.01.2020]

[B9] Ding B Nakamura K Kono Y Ho MJ Peng CI (2014) *Begonia jinyunensis* (Begoniaceae,section Platycentrum), a new palmately compound leaved species from Chongqing, China. Botanical Studies (Taipei, Taiwan) 55(1): 62–69. 10.1186/s40529-014-0062-6 PMC5430339 28510980

[B10] Ding QL Zhao WY Yin QY Ye HG Song HZ Liao WB (2018) *Begonia ehuangzhangensis* (sect. Diploclinium, Begoniaceae), a new species from Guangdong, China. Phytotaxa 381(1): 107–115. 10.11646/phytotaxa.381.1.14

[B11] Ding HB Maw MB Yang B Bouamanivong S Tan YH (2020) An updated checklist of *Begonia* (Begoniaceae) in Laos, with two new species and five new records. PhytoKeys 137: 187–301. 10.3897/phytokeys.138.46718 PMC6968974 31988613

[B12] Don D (1825) Prodromus Florae Nepalensis. J. Gale Press, London, 256 pp 10.5962/bhl.title.86 [accessed 20.01.2020]

[B13] Gagnepain MF (1919) Nouveaux *Begonia* d’Asie quelques synosymes. Bulletin du Muséum National d’Histoire Naturelle 15: 1–279.

[B14] Golding J (2004) *Begonia* U 3888 is *Begonia longiciliata* C.Y.Wu. The Begonian 71: 154–156.

[B15] Gu CZ Peng CI Turland NJ (2007) Begoniaceae In: Wu CY, Raven PH (Eds) Flora of China (Vol. 13). (Clusiaceae through Araliaceae). Science Press & Missouri Botanical Garden, Beijing & St. Louis, Missouri. http://flora.huh.harvard.edu/china/mss/volume13/Begoniaceae.pdf

[B16] Hance HF (1883) Three new Chinese begonias. Le Journal de Botanique 21(7): 202–203.

[B17] Hara H (1973) New or noteworthy flowering plants from eastern Himalaya. Shokubutsu Kenkyu Zasshi 48(12): 358–359.

[B18] He SZ Chen SW Chen WH Zhang RM Shui YM (2019) A new species of *Begonia* Linn. (Begoniaceae) in karst regions from Guizhou, China. Phytotaxa 409(1): 49–52. 10.11646/phytotaxa.409.1.7

[B19] Huang SH Shui YM (1994) New taxa of *Begonia* from Yunnan. Yunnan Zhi Wu Yan Jiu 16(4): 333–342.

[B20] Hughes Moonlight Jara-Muñoz Tebbitt Wilson Pullan (2015–Present) *Begonia* Resource Centre. http://padme.rbge.org.uk/begonia/ [accessed 24.06.2020]

[B21] Irmscher E (1921) Plantae novae sinenses, diagnosibus brevibus descriptae a Dr. Henry Handel-Mazzetti. Anzeiger der Akademie der Wissenschaften in Wien. Mathematische-naturwissenchaftliche Klasse 58: 24–27.

[B22] Irmscher E (1927) Beitrage zur kenntnis der ostasiatischen Begonien. Mitteilungen aus dem Institut für allgemeine Botanik in Hamburg 6(3): 343–360.

[B23] Irmscher E (1939) Die Begoniaceen Chinas und ihre Bedeutung fur die Frage der Formbildung in polymorphen Sippen. Mitteilungen aus dem Institut für Allgemeine Botanik in Hamburg 10: 431–557.

[B24] Irmscher E (1951) Some new Chinese species of *Begonia*. Notes from the Royal Botanic Garden Edinburgh 21(1): 35–45.

[B25] Kiew R (2004) *Begonia sizemoreae* Kiew (Begoniaceae), a handsome new *Begonia* from Vietnam. Gardens’ Bulletin (Singapore) 54(6): 95–100. https://biodiversitylibrary.org/page/435991921 [accessed 20.01.2020]

[B26] Koch K (1837) Drei neue Schiefblatter oder Begonien. Berliner Allgemeine Gartenzeitung 25(10): 1–76.

[B27] Ku TC (1999) Begoniaceae. In: Ku TC (Ed.) Flora Reipublicae Popularis Sinica (Vol. 52(1)). Science Press, Beijing. [in Chinese]

[B28] Léveillé H (1909) Decades plantarum novarum XVI, in Fedde. Repertorium Specierum Novarum Regni Vegetabilis 7(1–3): 20–23. 10.1002/fedr.19090070108 [accessed 20.01.2020]

[B29] Li JW Tan YH Wang XL Wang XW Jin XH (2018) *Begonia medogensis*, a new species of Begoniaceae from Western China and Northern Myanmar. PhytoKeys 103: 13–18. 10.3897/phytokeys.103.25392 PMC6037654 29997445

[B30] Li HZ Guan KY Lin CW Peng CI (2019) *Begonia qingchengshanensis* (sect. Reichenheimia, Begoniaceae), a new species from Sichuan, China. Phytotaxa 349(2): 197–200. 10.11646/phytotaxa.349.2.12

[B31] Moonlight PW Ardi WH Padilla LA Chung KF Fuller D Girmansyah D Hollands R Jara-Munoz A Kiew R Leong WC Liu Y Mahardika A Marasinghe LDK O’Connor M Peng C-I Perez AJ Phutthai T Pullan M Rajbhandary S Reynel C Rubite RR Sang J Scherberich D Shui YM Tebbitt MC Thomas DC Wilson HP Zaini NH Hughes M (2018) Dividing and conquering the fastest-growing genus: Towards a natural sectional classification of the mega-diverse genus *Begonia* (Begoniaceae). Taxon 67(2): 267–323. 10.12705/672.3

[B32] Putzey JAAH (1857) *Begonia rex*. In: Van Houtte L (Ed.) Flores des Serres et des Jardins de l’Europe 12: 141–146. http://biodiversitylibrary.org/page/27803796 [accessed 20.01.2020]

[B33] Shui YM Chen WH (2017) Begonia of China. Yunnan Publishing Group Corporation and Yunnan Science & Technology Press, Kunming, 285 pp.

[B34] Shui YM Huang SH (1999) Note on the genus *Begonia* from Yunnan. Yunnan Zhi Wu Yan Jiu 21(1): 11–23.

[B35] Smith JE (1805) Exotic Botany (Vol. 2). Taylor, London.

[B36] Standards IUCN Petitions Subcommittee (2019) Guidelines for using the IUCN Red List Categories and Criteria. Version 14. http://www.iucnredlist.org/documents/RedListGuidelines.pdf

[B37] Tebbitt MC (2005) Begonias: Cultivation, Identification and Natural History. Timber Press, Portland, 3 pp.

[B38] Tebbitt MC Guan KY (2002) Emended circumscription of *Begonia silletensis* (Begoniaceae) and description of new subspecies form Yunnan, China. Novon 12(1): 133–136. 10.2307/3393252

[B39] Tian DK (1999) Horticultural Study on *Begonia versicolor*. Kunming Institute of Botany, Chinese Academy of Sciences, Master Thesis, Kunming. [in Chinese with English abstract]

[B40] Tian DK Li JX Guan KY (2001) New *Begonia* varieties –‘Kunming Bird’, ‘Kang-er’ and ‘White Snow’. Yuan Yi Xue Bao 28(2): 186–186. [in Chinese with English abstract]

[B41] Tian DK Guan KY Li JX Xiang JY (2002) New varieties of *Begonia* – ‘Dabai’, ‘Jianlü’, ‘Meinü’ and ‘Zhongda’. Yuan Yi Xue Bao 29(1): 90–91. [Chinese with English Abstract]

[B42] Tian DK Li C Tong Y Fu NF Wu RJ (2017) Occurrence and characteristics of natural hybridization of *Begonia* in China. Biodiversity Science 25(6): 654–674. [in Chinese with English abstract] 10.17520/biods.2017050

[B43] Tian DK Xiao Y Tong Y Fu NF Liu QQ Li C (2018) Diversity and conservation of Chinese wild begonias. Plant Diversity 40(3): 75–90. 10.1016/j.pld.2018.06.002 30175289 PMC6114263

[B44] Tian DK Li C Yu XL Zhou JL Liu KM Shu JP Zhou XL Xiao Y (2019) A new tuberous *Begonia* species endemic to Danxia landforms in central China. Phytotaxa 407(1): 101–110. 10.11646/phytotaxa.407.1.12

[B45] Tong Y Tian DK Shu JP Xiao Y Wang BM Fu NF (2019) *Begonia yizhouensis*, a new species in Begonia sect. Coelocentrum (Begoniaceae) from Guangxi, China. Phytotaxa 407(1): 59–70. 10.11646/phytotaxa.407.1.9

[B46] Wang WG He XS Ma XD Lin YL Shi JP Gong QB Shen JY (2019a) *Pycnarrhena pleniflora* and *Begonia gemmipara*, two newly recorded species from China. Acta Botanica, Boreali-Occidentalia Sinica 39(3): 563–567. [in Chinese with English abstract]

[B47] Wang WG Lang XA Yang LL Wu H Zhang S-Z (2019b) *Begonia zhongyangiana*, a new species of *Begonia* (Begoniaceae) from western China. Phytotaxa 407(1): 51–58. 10.11646/phytotaxa.407.1.8

[B48] Wu CY Ku TC (1995) New taxa of the *Begonia* L. (Begoniaceae) from China. Acta Phytotaxonica Sinica 33(3): 251–280.

[B49] Yang ZZ Zhou SS Li ZH Wang J Chen WH Shui YM (2015) Two new records of *Begonia* L. (Begoniaceae) from China. Plant Diversity and Resources 37(4): 425–427. [in Chinese with English abstract] 10.7677/ynzwyj201514131

[B50] Yang NT Zhang Y He LJ Fan RY Wang C Wang YH (2018) Ethnobotanical study on traditional edible sour plants of Bai nationality in Dali area. Journal of Plant Resources and Environment 27(2): 93–100. [Chinese with English Abstract]

[B51] Yu T (1948) An enumeration of begonias of South Western China. Bulletin of the Fan Memorial Institute of Biology 1: 113–130.

